# Isolation of exosome-like nanovesicles from Yiqi Huoxue Jiedu decoction and their anti-ovarian cancer activity

**DOI:** 10.3389/fphar.2025.1680551

**Published:** 2025-10-15

**Authors:** Qing-Ya Song, Yong-Jia Cui, Wen-Ping Lu

**Affiliations:** Department of Oncology, China Academy of Chinese Medical Sciences Guang’anmen Hospital, Beijing, China

**Keywords:** Chinese medicine compound, exosome-like nanovesicles (ELNVs), decoction conditions, isolate, ovarian cancer

## Abstract

**Background:**

Ovarian cancer, the deadliest gynecologic malignancy, is marked by high recurrence and poor prognosis. Exosome-like nanovesicles (ELNVs) derived from decocted traditional Chinese medicine (TCM) formulas encapsulate key bioactive components, and show promising therapeutic efficacy with good biocompatibility and targeting ability. However, research on ELNV extraction methodologies remains limited. Yiqi Huoxue Jiedu Decoction (YQHXJDD), an empirically validated TCM formula for ovarian cancer, lacks systematic investigation into its ELNV isolation protocols and therapeutic mechanisms. This study took YQHXJDD as the research subject, with a focus on extracting ELNVs from this TCM formula and validating their anti-ovarian cancer activity.

**Methods:**

YQHXJDD was extracted using varied decoction protocols. ELNVs were enriched by gradient and ultracentrifugation, resuspended in phosphate-buffered saline, and were characterized by size and concentration using transmission electron microscopy (TEM) and nanoparticle tracking analysis (NTA). The number of ELNVs enriched per dose of YQHXJDD was calculated to screen the decoction method that maximized ELNV yield and aligned with clinical medication practices. The colloidal stability of YQHXJDD ELNVs was evaluated via zeta potential measurement, and the bioactive components of YQHXJDD ELNVs were identified using untargeted metabolomics and RNA sequencing. An ID8-Luc peritoneal tumor-bearing mouse model was established and randomized into three groups: model, YQHXJDD, and YQHXJDD ELNVs. Mice received oral gavage for 3 consecutive weeks. Tumor burden was monitored via *in vivo* small animal imaging. Immunofluorescence was used to quantify CD86, CD206, CD4, and CD8 fluorescence in omental tumors. Luminex xMAP® Multiplex Assay was used to detect 36 cytokines/chemokines in serum, and flow cytometry was used to analyze splenic immune cell proportions and activity.

**Results:**

The creation efficiency of ELNVs formed during decoction is the highest with the optimal protocol, which is identified for maximizing YQHXJDD ELNV yield and involves 40 min of high-heat treatment followed by an additional 30 min of low-heat treatment. Zetaview-based zeta potential analysis demonstrated that YQHXJDD ELNVs by this protocol had an average zeta potential of −35.52 mV, indicating favorable colloidal stability. Untargeted metabolomics analysis revealed “Lipids and lipid-like molecules” as the most abundant metabolite superclass (34.98% of total metabolites), while “Carboxylic acids and derivatives” represented the dominant subclass (11.66%). Regarding small RNAs, those enriched in YQHXJDD ELNVs were confirmed to be typical, functionally active small RNAs. Notably, miR8783 and other miRNAs exhibited significantly high expression, along with high conservation and abundance across samples, suggesting they might act as core regulators of YQHXJDD ELNV biological functions. In *in vivo* assessments, compared with the model group, YQHXJDD ELNVs significantly inhibited ovarian cancer growth and metastasis, increased tumor necrosis factor-α (TNF-α) levels in mouse peripheral blood, and positively regulated the M1/M2 macrophage ratio and CD4^+^/CD8^+^ T cell ratio in omental tissues (all *P* < 0.05). However, YQHXJDD ELNVs had no significant effect on the proportions of splenic effector T cells, natural killer (NK) cells, activated CD8^+^ T cells, and activated NK cells (all *P* ≥ 0.05).

**Conclusion:**

The decoction ELNVs isolated from YQHXJDD can inhibit the growth and dissemination of ovarian cancer. The miRNAs and lipid components encapsulated in these ELNVs are presumed to be the critical mediators of these anti-ovarian cancer effects.

## 1 Introduction

Cancer is a leading cause of death in developed nations and the second most common cause of death in developing countries, second only to cardiovascular diseases (Bray et al., 2018). Ovarian cancer stands as the most lethal gynecological malignancy: data from the National Cancer Center (2024) indicate that approximately 61,000 new cases are diagnosed annually in China, with around 32,000 associated deaths (Han et al., 2024). Characterized by an insidious onset, approximately 70% of ovarian cancer patients present with peritoneal metastasis at diagnosis. Even after achieving complete tumor remission via surgery and platinum-based chemotherapy, over half of patients experience relapse within 3 years, exacerbating treatment complexity and further reducing survival rates (Hurwitz et al., 2021). Targeting the metastatic microenvironment of ovarian cancer is therefore critical for inhibiting metastasis, preventing recurrence, and lowering mortality.

Yiqi Huoxue Jiedu Decoction (YQHXJDD), a therapeutic prescription for ovarian cancer, functions by dredging the qi movement of the triple burner, harmonizing zang-fu organ qi and blood, and clearing latent toxins. The YQHXJDD is a Traditional Chinese Medicine (TCM) prescription specifically designed for ovarian cancer of the qi deficiency and blood stasis type. Its formulation is derived from two classical TCM formulas, namely, Lichong Decoction (developed by Zhang Xichun, a renowned modern TCM physician, for the treatment of gynecological tumors) and Guizhi Fuling Pill (formulated by Zhang Zhongjing, an outstanding TCM master of the Eastern Han Dynasty, for managing gynecological disorders), with subsequent modifications and adjustments based on these two foundational formulas (Weng and Lu, 2020). Previous randomized controlled clinical trials have confirmed its ability to prolong progression-free survival in patients with advanced ovarian cancer (Li et al., 2020), with its antitumor efficacy further validated by both *in vivo* and *in vitro* experiments (Li et al., 2018; Wu, 2025). Traditional Chinese medicine formulations are plagued by drawbacks including complex compositions, time-consuming decoction procedures, and an unpleasant bitter taste. Notably, decoction of TCM formulations generates plant-derived exosome-like nanovesicles (ELNVs) can encapsulate the primary active components of the formulations and have been demonstrated to exert superior therapeutic effects (Cao et al., 2024; WenJing et al., 2022).

ELNVs are biological nanostructures ranging from 30 to 300 nm in size, characterized by a bilayer phospholipid architecture and a typical “cup-shaped” morphology (Feng et al., 2025). Their bilayer phospholipid vesicular microstructure enables effective drug encapsulation, facilitating absorption by enterocytes into the bloodstream, and thereby enhancing drug release efficiency and stability (Junyan et al., 2023). ELNVs are enriched in bioactive lipids, proteins, RNA, DNA, and various other biologically active molecules. They exhibit favorable stability and biocompatibility (Yi et al., 2025), with no toxicity being observed upon oral administration, thus serving as natural nanocarriers for drugs and exerting therapeutic effects through tumor cell targeting (Dad et al., 2021; Zhang et al., 2021; Cao et al., 2019).

In traditional Chinese herbal compound decoction, the bioactive components obtained from the co-decoction of multiple herbs differ from those derived from mixing of individual herbal decoctions (Wang et al., 2021). Furthermore, in clinical practice, the application of TCM formulations is more consistent with the clinical medication model guided by the TCM formula theory of “sovereign, minister, assistant, and guide” (Li et al., 2025). Therefore, ELNVs derived from Chinese herbal compound decoction integrate the holistic regulatory effects of the latter with the intercellular communication functions of exosomes, along with favorable active targeting properties (Wei et al., 2025; Peng et al., 2025; Zhang et al., 2024). This provides novel insights for the development of modern drug delivery systems for TCM, holding promise for enhancing the therapeutic efficacy of TCM.

Currently, differential centrifugation followed by ultracentrifugation for impurity removal is the widely accepted method for isolating and purifying high-purity ELNVs (Rutter and Innes, 2017; Zhao et al., 2024). However, existing literature predominantly focuses on ELNVs derived from single herbs, and research on optimal decoction processes for ELNVs derived from traditional Chinese herbal compound decoctions remains scarce (Du et al., 2019; Yu et al., 2020). In this study, YQHXJDD was used as the research object to explore the decoction process for ELNVs for ELNVs from this TCM compound decoction, with concurrent validation of its corresponding anti-ovarian cancer pharmacodynamics.

## 2 Experimental materials

### 2.1 Experimental cells and animals

ID8-Luc cells were obtained from Shanghai Fuheng Biotechnology Co., Ltd., with passage ranging from 10 to 12. Female C57BL/6 mice (6–8 weeks old, weighing 20 ± 2 g) of specific pathogen-free (SPF) grade were purchased from SPF Bioscience Co., Ltd. (Animal Production License No.: SCXK (Jing) 2024-0001).

### 2.2 Experimental medicines and reagents

Chinese herbal decoction pieces of YQHXJDD consisted of *Huangqi* (Astragalus membranaceus (Fisch.) Bunge, Fabaceae, 30 g, raw herb), *Gouqizi* (Lycium barbarum L., Solanaceae, 15 g, raw herb), *Baizhu* (Atractylodes macrocephala Koidz., Asteraceae, 15 g, raw herb), *Ezhu* (Curcuma zedoaria (Christm.) Roscoe, Zingiberaceae, 9 g, raw herb), *Dannanxing* (Arisaema erubescens (Wall.) Schott, Araceae, 12 g, raw herb), *Haizao* (Sargassum pallidum (Turner) C. Agardh, Sargassaceae, 15 g, raw herb), *Qingpi* (Citrus reticulata Blanco, Rutaceae, 6 g, raw herb), *Wuyao* (Lindera aggregata (Sims) Kosterm., Lauraceae, 6 g, raw herb), *Rougui* (Cinnamomum cassia (L.) J. Presl, Lauraceae, 6 g, raw herb), *Cuobiejia* (Trionyx sinensis Wiegmann, Trionychidae, 12 g, raw herb), *Quanxie* (Buthus martensii Karsch, Buthidae, 5 g, raw herb), *Baihuasheshecao* (Hedyotis diffusa Willd., Rubiaceae, 15 g, raw herb), *Qinghao* (Artemisia annua L., Asteraceae, 12 g, raw herb), *Maozhaocao* (Ranunculus ternatus Thunb., Ranunculaceae, 15 g, raw herb), *Shancigu* (Tulipa edulis (Miq.) Baker, Liliaceae, 12 g, raw herb). All herbs were obtained from Hebei Zhijia Pharmaceutical Co., Ltd. Phosphate-buffered saline (PBS) was purchased from Sangon Biotech (Cat. No.: E607008). 0.45 μm filter membrane was purchased from JieTe (Cat. No.: FPV403030). Fluorescein potassium salt was purchased from Fubaike (Cat. No.: DCM9513). Uranyl acetate was purchased from Zhongjing Keyi (Cat. No.: 1722586). Citrate buffer (pH 6.0) was purchased from Zhongshan Jinqiao (Cat. No.: ZLI-9064). DAPI was purchased from Beyotime (Cat. No.: EPX360-26092-901). Anti-fluorescence quenching mounting medium was purchased from MDL (Cat. No.: 30093360).

Cytokine and Chemokine 36-Plex Mouse ProcartaPlex™ Panel 1A was purchased from Thermo Fisher Scientific (Cat. No.: C1005). Antibody diluent was purchased from Boster (Cat. No.: AR1016). Reagents and antibodies used in flow cytometry included Stain Buffer (BD, Cat. No.: 554657), Zombie (Biolegend, Cat. No.: 423102), membrane-breaking agent (eBioscience, Cat. No.: 00-5523-00), RPMI-1640 (Procell, Cat. No.: PM150110B), Leukocyte Activation Cocktail (BD, Cat. No.: 550583), Mouse CD45 APC-CY7 (BD, Cat. No.: 557659), Mouse CD49b APC (BD, Cat. No.: 560628), Mouse Granzyme B PE-CY7 (Biolegend, Cat. No.: 372214), Mouse IFN-r PE (Biolegend, Cat. No.: 505808), Mouse CD163 BV421 (Biolegend, Cat. No.: 155309), Mouse F4/80 APC (Biolegend, Cat. No.: 123116), Mouse CD86 PE-CF594 (Biolegend, Cat. No.: 105042), Mouse CD8 PercP (BD, Cat. No.: 553036), Mouse CD25 PE (BD, Cat. No.: 554866), Mouse CD4 FITC (Biolegend, Cat. No.: 100510), Mouse CD11b BV605 (Biolegend, Cat. No.: 101257).

### 2.3 Experimental equipment

The experimental equipment included pipettes (Eppendorf, Cat. No.: Research Plus), a small refrigerated centrifuge (Beckman, Cat. No.: Microfuge 20R), a refrigerated centrifuge (Heal Force, Cat. No.: Neofuge1600R), an ultra-low temperature freezer (Thermo Fisher Scientific, Cat. No.: 905), an ultracentrifuge tube (Beckman Coulter, Cat. No.: 355618), an ultracentrifuge (Hitachi, Cat. No.: CP100MX, rotor: Type 70Ti), a transmission electron microscope (Hitachi, Cat. No.: HT-7700), a nanoparticle size analyzer (NanoFCM, Cat. No.: N30E), a dehydrator (Kehai, Cat. No.: KH-TK), an embedding machine (Wuhan Junjie, Cat. No.: JB-L5), a paraffin microtome (Leica, Cat. No.: RM2235), a fluorescence microscope (Nikon, Cat. No.: ECLIPSE-CI), a small animal *in vivo* imaging system (VISQUE, Cat. No.: Invivo Smart-LF), BD Fortessa flow cytometer (BD, LSRFortessa) and Luminex™ 200™ Instrument System (Thermo Fisher Scientific, Cat. No.: APX10031), 20 μL–300 μL multichannel pipette (Eppendorf, Cat. No.: 3123000306), 2 μL–1000 μL single-channel pipette (Eppendorf, Cat. No.: 3123000268), Hand-Held Magnetic Plate (Thermo Fisher Scientific, Cat. No.: EPX-55555-000), Vortex mixer (ZX4 Advanced IR, VELP Scientifica Srl, Cat. No.: SA202A0280), Microplate shaker (capable of reaching a rotation speed of 500 rpm) (Kylin-Bell lab Instruments, Cat. No.: QBQ-9001), Refrigerated centrifuge suitable for 1.5–2 mL centrifuge tubes (Xiangyi, Cat. No.: H1750R), Nanoparticle tracking analyzer (PARTICLE METRIX, Cat. No.: PMX120), Small Benchtop Refrigerated Centrifuge (Beckman, Cat. No.: Microfuge 20R).

## 3 Methods

### 3.1 Exploration of the extraction process of YQHXJDD ELNVs

**FIGURE 1:**
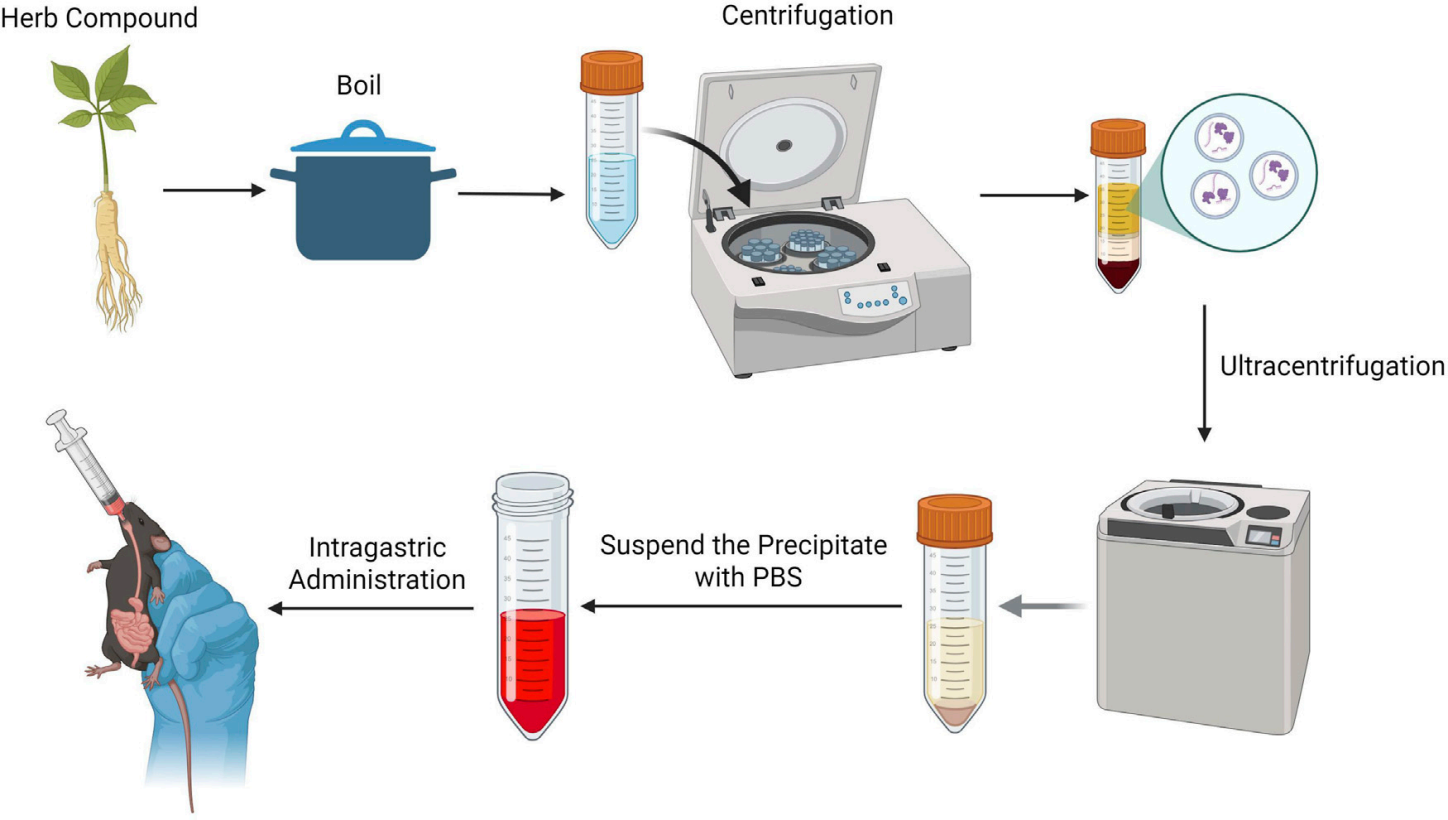
Experimental procedure.

The research strategy of this study was as follows: Initially, various decoction protocols for YQHXJDD were established to explore the optimal extraction process for maximizing the yielded of YQHXJDD-derived ELNVs. Subsequently, the extracted ELNVs underwent purification and precipitation via differential centrifugation and ultracentrifugation. Following resuspension in PBS, the resultant preparation was administered to mice through gavage, aiming to investigate the underlying pharmacodynamic mechanisms. A schematic overview of the experimental workflow is provided in Figure 1.

#### 3.1.1 Preparation for YQHXJDD

Chinese herbal decoction pieces of YQHXJDD were soaked in deionized water for 40 min. Subsequently, six different decoction schemes were employed, as shown in Table 1.

**TABLE 1:** Chinese herbal decoction protocols.

Decoction scheme	Volume of deionized water (L)	Boiling time on high heat (min)	Simmering time on low heat (min)
Scheme 1	1	40	10
Scheme 2	1	40	20
Scheme 3	1	40	30
Scheme 4	1	40	35
Scheme 5	1.2	40	40
Scheme 6	1.2	40	45

#### 3.1.2 Centrifugation of YQHXJDD ELNVs

The herbal medicine solution was processed using gradient centrifugation and ultracentrifugation with the following steps:

1. Thawed the sample at 37 °C with gentle agitation.
2. Transferred the sample to a new centrifuge tube and centrifuged at 2,000 × g for 30 min at 4 °C.
3. Transferred the supernatant to a fresh tube and recentrifuged at 10,000 × g for 45 min at 4 °C to remove larger vesicles.
4. Collected the supernatant and filtered through 0.45-μm membranes to obtain the filtrate.
5. Transferred the filtrate to an ultracentrifuge tube. Using an appropriate rotor, ultracentrifuged at 100,000 × g for 70 min at 4 °C.
6. Discard the supernatant, resuspend the pellet in 10 mL of pre-cooled 1× PBS, and recentrifuge at 100,000 × g for 70 min at 4 °C using the same rotor.
7. Discarded the supernatant, resuspended the pellet in 10 mL of pre-cooled 1× PBS.

Aliquot 20 μL for TEM analysis, 10 μL for particle size determination, and store the remaining exosome-enriched fraction at −80 °C.

#### 3.1.3 Transmission electron microscopy (TEM) observation of YQHXJDD ELNVs

YQHXJDD ELNVs were visualized using transmission electron microscopy (TEM) following the protocol below:

1. A 10 μL aliquot of the YQHXJDD ELNV sample was dropped onto a copper grid and allowed to settle for 1 min.
2. Excess liquid was blotted off with filter paper.
3. The copper grid was stained with 10 μL of 2% uranyl acetate (w/v) for 1 min.
4. Residual stain was removed with filter paper, and the grid was air-dried at room temperature for 5–10 min.
5. TEM imaging was performed at an accelerating voltage of 80 kV to acquire micrographs.

#### 3.1.4 Particle size and concentration analysis of YQHXJDD ELNVs

The particle size and concentration of YQHXJDD ELNVs were determined using a nanoparticle size analyzer (NanoFCM, Cat. No.: N30E), with results validated by nanoparticle tracking analysis (NTA). A 10 μL aliquot of ELNVs isolated from YQHXJDD was diluted to at a ratio of 500:1 with phosphate-buffered saline (PBS) (pH 7.4). Prior to sample analysis, the instrument was calibrated with a standard 100 nm polystyrene standard beads to confirm its performance. Gradient dilution was conducted to prevent clogging of the injection needle. Following sample analysis, data on the particle size distribution and concentration of the ELNVs were generated via the instrument.

#### 3.1.5 Zeta potential measurement

YQHXJDD ELNV samples were diluted at a ratio of 1:250,000 using ultrapure water. Environmental Parameters: Sensed temperature of 25.64 °C, adjusted pH of 7.0 (maintained constant during detection), and subsequently analyzed for zeta potential using a NTA. The instrument model was ZetaVIEW (Serial Number: 20–602), equipped with ZetaView software (Version 8.05.14 SP7). The optical parameters employed included a laser with a wavelength of 520 nm, complemented by a scatter filter for efficient signal collection. All experiments strictly adhered to the standard operating procedure (SOP) designated as “ZP_EV”, wherein 11 specific positions were selected for comprehensive profile scanning to ensure representative data acquisition. For particle screening, stringent criteria were applied: particles with a maximum area of 1,000, minimum area of 5, and minimum brightness of 20 were retained, effectively eliminating background noise and irregular particles to guarantee the quality and reliability of the analyzed particle population.

#### 3.1.6 Untargeted metabolomics analysis

A total of 6 YQHXJDD samples were used for untargeted metabolomics analysis. Samples were slowly thawed at 4 °C, and metabolites were extracted by adding pre-cooled methanol/acetonitrile/water (2:2:1, v/v/v). After vortex mixing, the mixture was subjected to low-temperature sonication for 30 min and then incubated at −20 °C for 10 min. Following centrifugation at 14,000 × g for 20 min at 4 °C, the supernatant was collected and vacuum-dried. For mass spectrometry (MS) analysis, the dried residue was reconstituted with 100 μL of acetonitrile/water (1:1, v/v), vortexed thoroughly, and centrifuged again at 14,000 × g for 15 min at 4 °C. The resulting supernatant was collected for injection. Chromatographic separation was performed on an Agilent 1,290 Infinity Liquid Chromatography (LC) system and a Thermo Scientific Vanquish Ultra-High Performance Liquid Chromatography (UHPLC) system, both equipped with a Waters ACQUITY UPLC BEH Amide column (2.1 mm × 100 mm, 1.7 μm) maintained at 25 °C. The mobile phase consisted of Phase A (water containing 25 mM ammonium acetate and 25 mM ammonia) and Phase B (acetonitrile), with a flow rate of 0.5 mL/min and an injection volume of 2 μL. The gradient elution program was set as follows: 95% Phase B from 0 to 0.5 min; linear decrease of Phase B to 65% from 0.5 to 7 min; further decrease to 40% from 7 to 8 min; maintenance at 40% from 8 to 9 min; linear increase back to 95% from 9 to 9.1 min; and maintenance at 95% from 9.1 to 12 min. Samples were stored in a 4 °C autosampler, and all analyses were conducted in a random order. Quality Control (QC) samples, prepared by mixing equal volumes of all sample extracts, were inserted to monitor the stability of the analytical system. MS detection was carried out using an AB Sciex Triple TOF 6600 mass spectrometer operated in both positive and negative electrospray ionization (ESI) modes. ESI source parameters: Nebulizer/auxiliary heater gas 1 (Gas1) = 60; auxiliary heater gas 2 (Gas2) = 60; curtain gas (CUR) = 30 psi; ion source temperature = 600 °C; spray voltage (ISVF) = ±5500 V (for positive and negative modes, respectively). Mass scanning parameters: Primary mass-to-charge ratio (m/z) detection range = 60–1,000 Da with a scan accumulation time of 0.20 s/spectra; secondary product ion m/z detection range = 25–1,000 Da with a scan accumulation time of 0.05 s/spectra. Secondary MS acquisition settings: Data were acquired via data-dependent acquisition (IDA) mode with peak intensity screening. Declustering potential (DP) = ±60 V (for positive and negative modes, respectively); collision energy = 35 ± 15 eV. For IDA, the dynamic exclusion range for isotope ions was 4 Da, and 10 fragment spectra were collected per scan. Raw data were converted to mzXML format using ProteoWizard software, followed by peak alignment, retention time correction, and peak area extraction using XCMS software. Data preprocessing included three sequential steps: (1) filtering out ions with >50% missing values; (2) imputing missing values for the remaining features using the k-Nearest Neighbors (KNN) algorithm; (3) removing features with a Relative Standard Deviation (RSD) > 50%. Subsequently, metabolite identification was performed by matching with an in-house database, with the matching criteria of mass error <10 ppm, consistent retention time (RT), and matched tandem mass spectrometry (MS/MS) spectra.

#### 3.1.7 RNA sequencing for YQHXJDD ELNVs

miRNA library construction was performed using the QIAseq® miRNA Library Kit (Qiagen, Cat. No. 331505) following the manufacturer’s instructions with minor modifications. All operations were conducted in a Heal Force biosafety cabinet (Model: HFsafe-1500LC) to prevent contamination, and Eppendorf Research Plus single-channel pipettes were used for accurate reagent dispensing. For 3′ adapter ligation, a 20 μL reaction mixture was prepared in a PCR tube, containing 10 ng of input RNA, Nuclease-Free Water (Invitrogen, Cat. No. AM9930), QIAseq 3′ Adapter, QIAseq RI, QIAseq 3′ Ligase, QIAseq 3′ Buffer, and 2× miRNA Ligation Activator. The mixture was incubated in a Thermo ProFlex PCR System at 28 °C for 60 min, followed by 65 °C for 20 min, and finally held at 4 °C for 5 min. The reaction product was immediately used for the next step without storage. Subsequently, 5′ adapter ligation was performed by adding Nuclease-Free Water, QIAseq 5′ Buffer, QIAseq RI, QIAseq 5′ Ligase, and QIAseq 5′ Adapter to the 3′ ligation product, with the total volume adjusted to 40 μL. The reaction was carried out in the PCR System at 28 °C for 30 min, 65 °C for 20 min, and then held at 4 °C. For cDNA synthesis, QIAseq miRNA NGS RT Initiator was first added to the 5′ ligation product (total volume 42 μL), followed by a gradient temperature program: 75 °C for 2 min, 70 °C for 2 min, 65 °C for 2 min, 60 °C for 2 min, 55 °C for 2 min, 37 °C for 5 min, 25 °C for 5 min, and finally held at 4 °C. Thereafter, QIAseq miRNA NGS RT Primer, Nuclease-Free Water, QIAseq miRNA NGS RT Buffer, QIAseq miRNA NGS RI, and QIAseq miRNA NGS RT Enzyme were added to adjust the system volume to 60 μL. The mixture was incubated at 50 °C for 60 min, 70 °C for 15 min, and 4 °C for 5 min cDNA purification was performed using QMN Beads: 143 μL of beads was added to the reverse transcription product, vortexed, and incubated at room temperature for 5 min. The mixture was then placed on a magnetic stand to remove the supernatant. The beads were washed twice with 200 μL of freshly prepared 80% ethanol, dried for 10 min, and eluted with 17 μL of NF-Water. A total of 15 μL of the eluate was collected as purified cDNA and stored at −20 °C if not used immediately. Library amplification was conducted in an 80 μL system containing purified cDNA, Nuclease-Free Water, QIAseq miRNA NGS Library Buffer, HotStarTaq DNA Polymerase, Forward Primer, and Index Primer. The PCR program was set as follows: initial denaturation at 95 °C for 15 min; followed by cycles of denaturation at 95 °C for 15 s, annealing at 60 °C for 30 s, and extension at 72 °C for 15 s (the number of cycles was adjusted based on RNA input, typically 15–20 cycles); and a final extension at 72 °C for 2 min, then held at 4 °C. Library sorting was performed using QMN Beads: 75 μL of beads was mixed with 75 μL of the amplification product, incubated, and the supernatant was transferred to a new tube. Then, 130 μL of beads was added to the supernatant, followed by incubation, magnetic separation, ethanol washing, drying, and elution with 17 μL of NF-Water. A total of 15 μL of the eluate was collected as the final miRNA library.

The concentration of the library was determined using a Promega Quantus Fluorometer (Model: E6150), and the fragment size was analyzed with a Bioptic Qsep100 Automatic Nucleic Acid and Protein Analysis System. The libraries were stored at −20 °C before sequencing. The adapter sequence of the miRNA library was 5′-AACTGTAGGCACCATCAATNNNNNNNNNNNNAGATCGGAAGAGCACACGTCTGAACTCCAGTCAC-3′, which contained a Unique Molecular Index (UMI) for subsequent data analysis. After library construction, the small RNA sequencing library was sequenced using the paired-end 150 (PE150) sequencing strategy. FastQC software was first used to evaluate the quality of the sequencing library and assess the overall sequencing data quality. Then, fastp software was employed for raw sequence preprocessing, including the removal of N bases at both ends of the sequences, filtering of sequences with a Phred quality score (Q) below 20, and excision of residual adapter sequences to obtain clean reads. For further data refinement, the clean reads were aligned to the Rfam database using the Bowtie short-read alignment tool to filter out non-coding RNAs (ncRNAs) such as ribosomal RNA (rRNA) and transfer RNA (tRNA). Finally, the remaining reads were aligned to the reference genome using Bowtie for subsequent miRNA identification and analysis.

### 3.2 Pharmacological evaluation of YQHXJDD ELNVs

#### 3.2.1 Experimental materials

YQHXJDD herbal pieces were decocted using Protocol 3: soaked in deionized water for 40 min, decocted over high heat for 40 min then low heat for 30 min, yielding ∼250 mL of decoction. Post-decoction, ultracentrifugation was performed; the precipitate was resuspended in PBS to a YQHXJDD ELNVs concentration of 7.5 × 10^10^ particles·mL^-1^.

#### 3.2.2 Animal grouping and tumor model establishment

Mice were randomly assigned to three groups: control tumor-bearing (model group), YQHXJDD, and YQHXJDD ELNVs. Sample size calculation was performed based on the effect size derived from the pilot study. ID8-Luc cells in logarithmic growth phase were harvested, resuspended in sterile phosphate-buffered saline (PBS, pH 7.4) at 1 × 10^7^ cells·mL^-1^, and maintained on ice. Following a 7-day acclimatization period, each mouse received an intraperitoneal injection of 500 μL ID8-Luc cell suspension (5 × 10^6^ cells) to induce peritoneal dissemination of epithelial ovarian cancer.

The Ethic Committee of China Academy of Chinese Medical Sciences Guang’anmen Hospital has approved the ethical and scientific application of all animal experiments in our study. The study was strictly conducted in accordance with “Guide for the care and use of Laboratory animals” published by the National Institute of Health.

#### 3.2.3 Treatment administration

Two weeks post-modeling, mice were randomized into three groups (n = 6 per group, ear-tagged) and treatments were administered via oral gavage for 21 consecutive days at a dosage of 20 μL/g body weight. The model control group was given PBS by gavage at a dose of 20 μL/g body weight, once daily for 21 consecutive days. The YQHXJDD ELNVs group received exosomes extracted by Decoction Protocol 3 and resuspended in PBS via gavage at a dose of 20 μL/g body weight, once daily for 21 consecutive days. The YQHXJDD group was administered the herbal decoction prepared from YQHXJDD by gavage at a dose of 20 μL/g body weight, once daily for 21 consecutive days.

#### 3.2.4 Tumor burden assessment via luminescence imaging

Tumor burden was evaluated based on luminescence of the ID8-luc cells detected by an *in vivo* small animal imaging system. Each mouse was intraperitoneally injected with 100 μL of luciferin substrate (20 mg/mL). After injection, the mice were allowed to move freely for 5 min to facilitate substrate distribution. Subsequently, the animals were placed in a closed chamber and anesthetized with 3% isoflurane in oxygen at a flow rate of 0.5 L/min. At 10 min post-luciferin administration, once a stable anesthetic state was confirmed (evidenced by the absence of corneal reflex and muscle relaxation), the mice were transferred to the temperature-controlled stage of the *in vivo* imaging system. Anesthesia was maintained with 1%–2% isoflurane delivered via a nasal cone throughout the imaging process, and bioluminescent signals emitted by ID8-Luc cells were continuously monitored and recorded.

Mice in each group were subjected to observation of tumor tissue growth on the day before administration, as well as on days 7, 14, and 21 after the initiation of administration.

#### 3.2.5 Immunofluorescence staining

Omental tumor tissues were processed through embedding, sectioning, dewaxing, and staining procedures. Fluorescence intensities of CD206, CD86, CD4, and CD8 were quantified using a fluorescence microscope.

#### 3.2.6 Flow cytometry analysis

Splenic cell subpopulations were analyzed by flow cytometry across all groups. The evaluated parameters included: the percentage of M1 macrophages within the total macrophage population, the proportion of effector T cells among total T cells, the ratio of activated natural killer (NK) cells in non-T immune cells, and the expression of functional markers (Granzyme B and IFN-γ) in both splenic CD8^+^ T cells and NK cells. The procedures were as follows:

1. Mouse spleens were retrieved from the preservation medium and transferred onto a 70 μm cell strainer placed over a 50 mL centrifuge tube. Tissues were gently disrupted using a pestle, and the resulting cell suspension was rinsed and resuspended in 5 mL of complete RPMI 1640 medium.
2. Cell counts were determined, and the cell concentration was adjusted to 1 × 10^7^ cells/mL.
3. A 100 μL aliquot of the cell suspension was dispensed into a test tube, followed by the addition of Zombie dye (Biolegend, Cat. No.: 423102) at the manufacturer-recommended volume. The mixture was vortexed briefly to ensure homogeneity and incubated for 15 min in the dark.
4. After incubation, 1 mL of PBS was added, and the suspension was vortexed gently. Cells were pelleted by centrifugation at 1,500 rpm for 5 min, and the supernatant was carefully aspirated to enrich the cell pellet.
5. The corresponding antibodies were added at their respective recommended doses, and the mixture was vortexed gently before being incubated for 15 min in the dark.
6. Subsequently, 1 mL of PBS was added, and the suspension was vortexed gently. Cells were pelleted again by centrifugation at 1,500 rpm for 5 min, with the supernatant aspirated to further enrich the cell pellet.
7. Finally, 200 μL of PBS was added to resuspend the cells via gentle vortexing, and the samples were processed for flow cytometric analysis.

Detection of intracellular Granzyme B and IFN-γ was performed using the following protocol, which built upon the procedures described in steps (1) and (2).

8. A 500-μL aliquot of the cell suspension was stimulated with a PMA-containing cocktail (50 ng/mL PMA, 1 μg/mL ionomycin, and 1× GolgiStop™) for 4 h.
9. Following centrifugation at 1,500 rpm for 5 min, the supernatant was discarded, and the cell pellet was resuspended in 100 μL of staining buffer. Zombie dye was added at the manufacturer’s recommended concentration, and the mixture was incubated for 15 min in the dark with gentle vortexing.
10. Cells were washed with 1 mL of PBS, centrifuged at 1,500 rpm for 5 min, and the supernatant was discarded.
11. Fluorochrome-conjugated antibodies against CD45, CD3, CD49b, and CD8 were added at optimized dilutions, followed by a 15-min incubation in the dark.
12. Cells were fixed and permeabilized using 1 mL of 1× Fixation/Permeabilization Buffer for 15 min in the dark.
13. After washing with 1 mL of 1× Permeabilization Buffer, cells were pelleted by centrifugation at 1,500 rpm for 5 min.
14. Intracellular staining was performed by incubating cells with antibodies against Granzyme B and IFN-γ at recommended concentrations for 30 min in the dark.
15. Cells were washed once more with Buffer, centrifuged, and resuspended in an appropriate volume for flow cytometric analysis.

#### 3.2.7 Luminex xMAP® Multiplex Cytokine Assay

A high-throughput liquid protein chip analysis method was used to detect 36 cytokines in mouse peripheral blood. The procedures were as follows: reconstitution of standards, 4-fold serial dilution of standards, washing of microspheres, addition of samples for incubation, plate washing, addition of detection antibodies, plate washing, addition of streptavidin-phycoerythrin, plate washing, and on-machine detection.

#### 3.2.8 Statistical analysis

Data were analyzed using SPSS 25.0 statistical software. All results were presented as mean ± standard deviation (x̄ ± s). Comparisons among the three groups were performed using one-way analysis of variance (ANOVA). If ANOVA indicated a significant overall difference (*P* < 0.05), Tukey’s Honestly Significant Difference (HSD) post-hoc test was further used for pairwise comparisons. A p-value <0.05 was considered statistically significant. For the Luminex xMAP® Multiplex Cytokine Assay, a five-parameter nonlinear regression method was used to fit the standard curve, and the concentration values were calculated accordingly.

## 4 Results

### 4.1 Transmission electron microscopy results of YQHXJDD ELNVs

**FIGURE 2:**
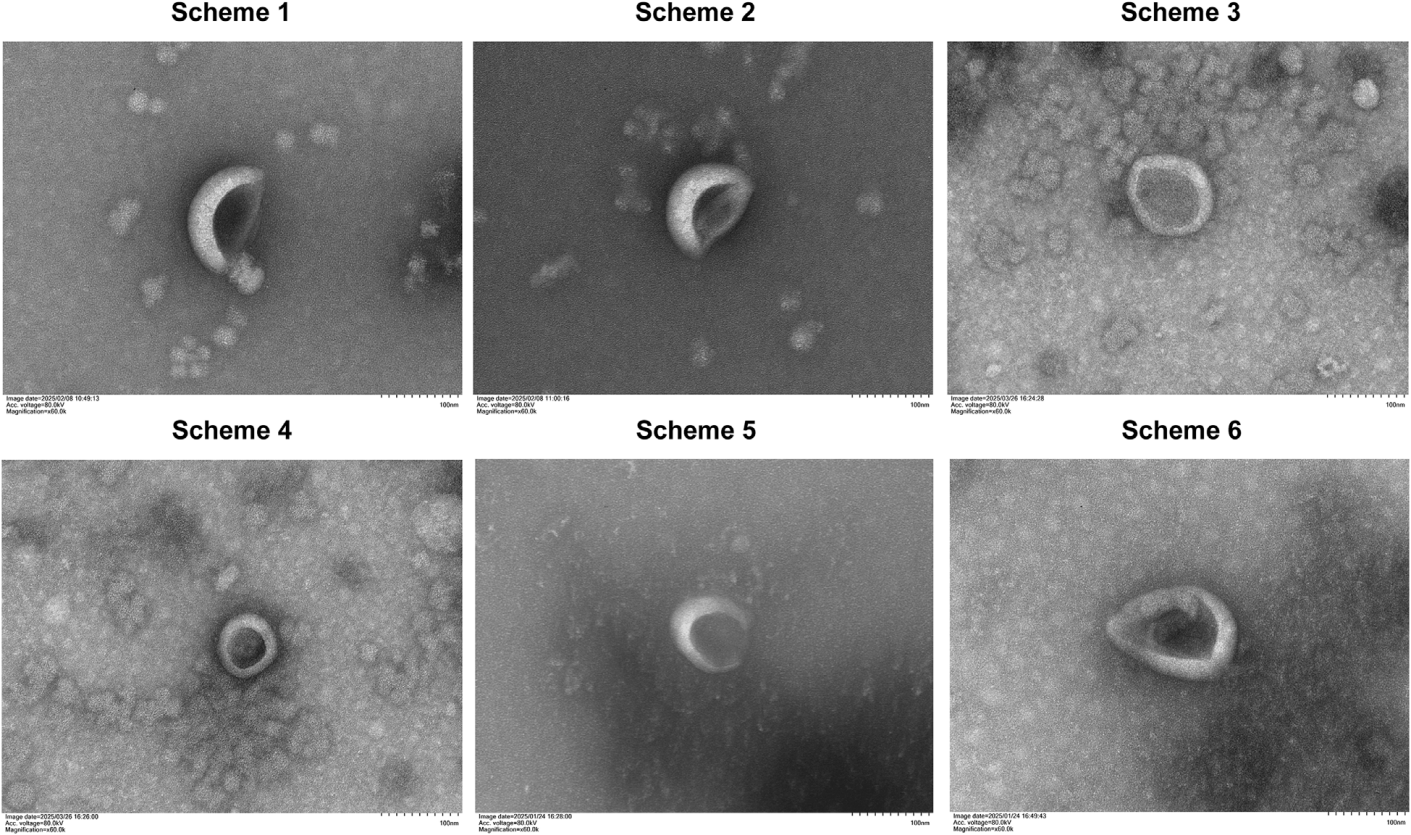
Morphology of YQHXJDD ELNVs by transmission electron microscopy.

ELNVs isolated from YQHXJDD via all six decoction protocols exhibited the characteristic “cup-shaped” morphology (Figure 2).

### 4.2 Volume and concentration of YQHXJDD ELNVs

The six decoction protocols yielded varying volumes of YQHXJDD. Each decoction was thoroughly mixed, and aliquots were subjected to ultracentrifugation to collect YQHXJDD ELNV pellets. The pellets were resuspended in PBS, and 10 μL of the resuspension was used for particle size and concentration analysis. The total number of ELNVs per dose of herbal material was calculated as follows: ELNV concentration (from resuspended pellets) × PBS resuspension volume (Table 2), normalized to the decoction yield per dose of herbal material. Results indicated that Decoction Protocol 3 yielded the highest total number of ELNVs after ultracentrifugation, with 7.504 × 10^13^ ELNVs per dose of herbal material.

**TABLE 2:** Particle size and concentration detection.

Sample	PBS volume	Average particle size (nm)	Concentration (Particles/mL)	Number of ELNVs per dose of YQHXJDD
Protocol 1	500 μL	73.3	1.02*10^11^	5.1*10^12^
Protocol 2	400 μL	72.0	2.77*10^11^	1.108*10^13^
Protocol 3	560 μL	71.1	1.34*10^12^	7.504*10^13^
Protocol 4	500 μL	71.3	7.34*10^11^	3.67*10^13^
Protocol 5	500 μL	78.8	9.31*10^10^	4.655*10^12^
Protocol 6	500 μL	73.5	4.65*10^10^	2.325*10^12^

### 4.3 Zeta potential characteristics

**FIGURE 3:**
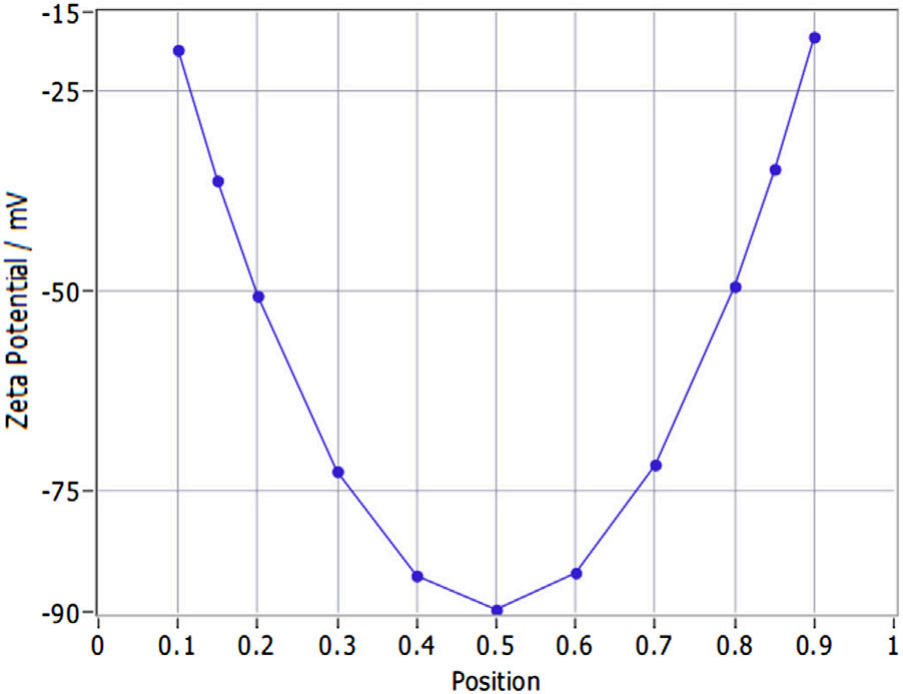
Zeta potential variation with stationary layer position in YQHXJDD ELNVs.

The corrected zeta potential of the samples at 25 °C was −35.52 ± 1.13 mV (mean ± SD), calculated using the Smoluchowski model (ZP factor = 12.6). Regarding the zeta potential distribution, its central value was consistent with the aforementioned corrected zeta potential (−35.52 mV), and the full width at half maximum (FWHM) was measured as 3.57 mV (Figure 3). This narrow FWHM value indicates a uniform surface charge distribution across the nanoparticle population, which is a key indicator of nanoparticle colloidal stability. For stationary layer (SL) potentials: SL1 (position 0.149 mm) was −36.112 mV, SL2 (position 0.851 mm) was −34.666 mV, with a potential difference (∆SL) of 1.45 mV. This small ∆SL indicates stable detection system performance, excluding systematic errors in zeta potential measurements. The nanoparticles’ electrophoretic mobility was −2.81 ± 0.09 μm/s/V/cm, corrected to −2.77 μm/s/V/cm at 25 °C. This corrected value provides a more accurate reference for correlating with zeta potential and evaluating nanoparticle electrophoretic behavior in aqueous environments.

### 4.4 Untargeted metabolomics analysis in YQHXJDD ELNVs

**FIGURE 4:**
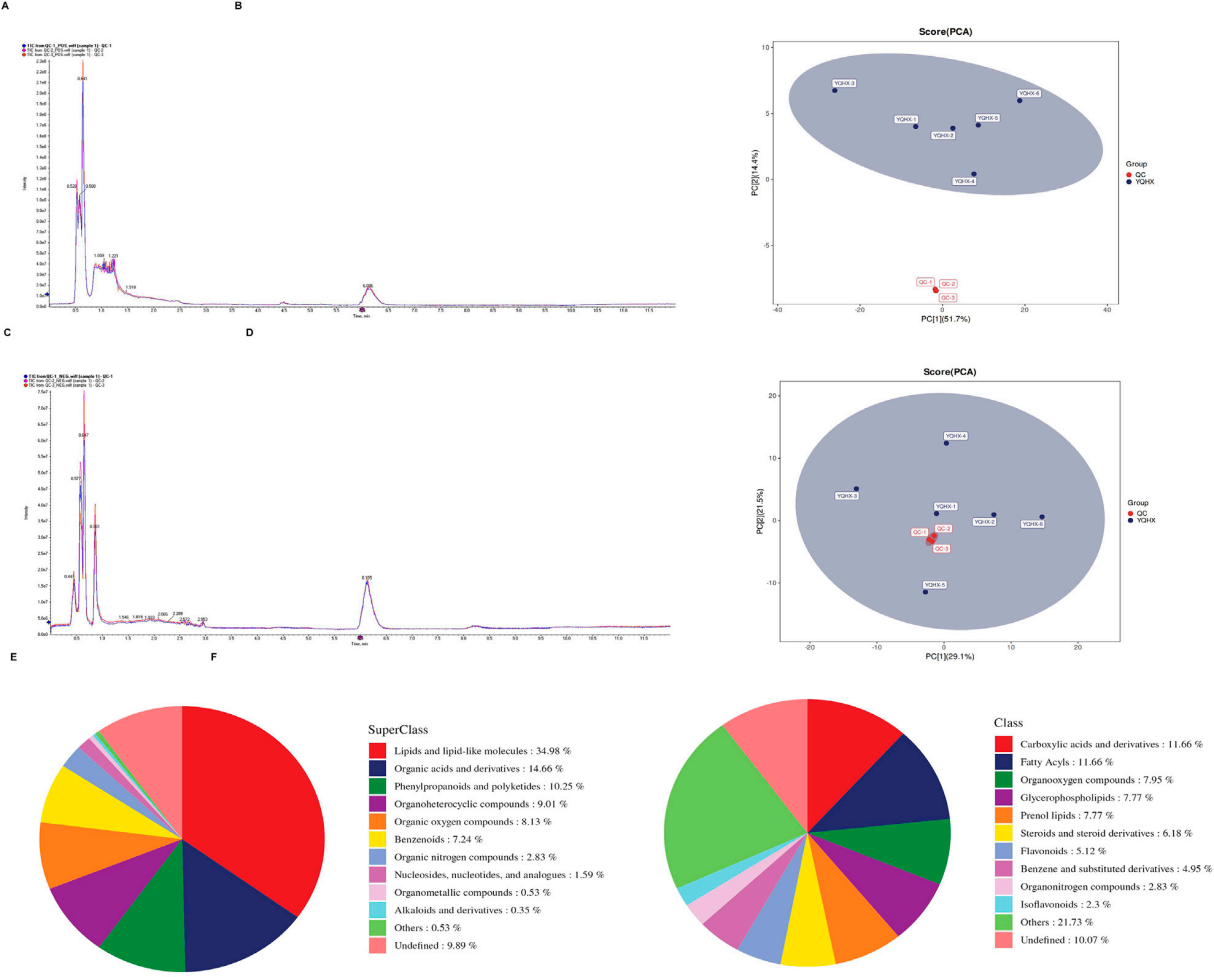
Untargeted Metabolomics Analysis of YQHXJDD ELNVs. **(A)** Overlaid Total Ion Chromatograms (TICs) of QC samples in positive ion mode; **(B)** Principal Component Analysis (PCA) score plot of all samples in positive ion mode; **(C)** Overlaid Total Ion Chromatograms (TICs) of QC samples in negative ion mode; **(D)** Principal Component Analysis (PCA) score plot of all samples in negative ion mode; **(E)** Class classification of metabolites from YQHXJDD ELNVs; **(F)** SuperClass classification of metabolites from YQHXJDD ELNVs.

In the untargeted metabolomics analysis, multivariate statistical analysis was first employed to construct the metabolic profiles of the study samples. Total Ion Chromatograms (TICs) demonstrated good chromatographic separation for QC samples. Notably, the overlaid TICs of different QC samples showed high consistency in response intensity and retention time, indicating reliable stability of the chromatographic system (Figures 4A,C). Subsequently, the Principal Component Analysis (PCA) score plots of the samples in the dataset were analyzed. Under both positive and negative ion modes, all QC samples exhibited obvious clustering (Figures 4B,D), which clearly confirmed the stability and reproducibility of the analytical system. Finally, all metabolites identified in this study (combining those identified in positive and negative ion modes) were statistically classified based on their Chemical Taxonomy assignment information. Figures 4E,F showed the proportion of metabolite quantities in the SuperClass and Class classifications of YQHXJDD ELNVs metabolites, respectively. In the SuperClass classification, “Lipids and lipid-like molecules” accounted for the largest proportion (34.98%), while in the Class classification, “Carboxylic acids and derivatives” had the highest proportion (11.66%).

### 4.5 Small RNA sequencing and expression in YQHXJDD ELNVs

**FIGURE 5:**
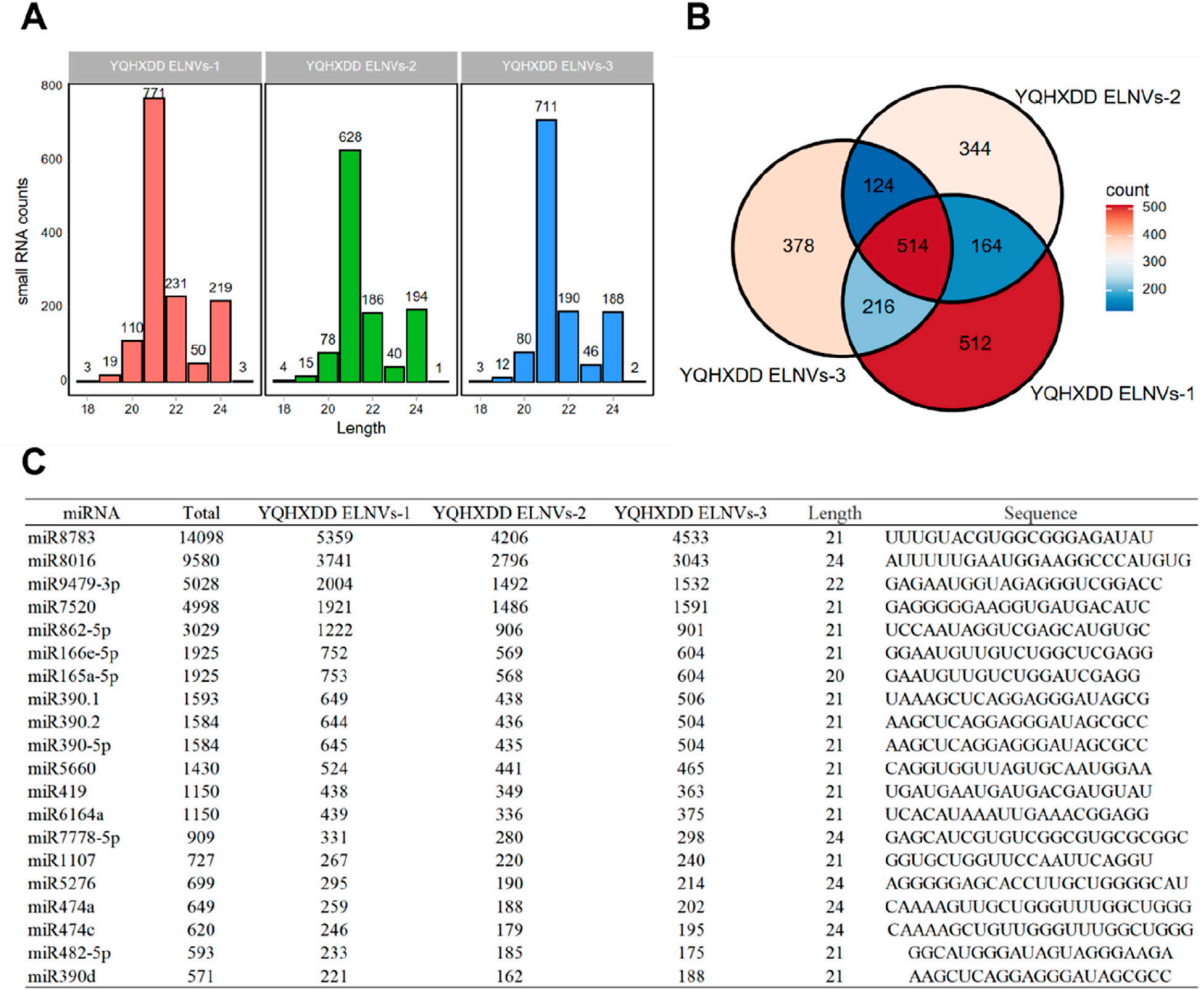
miRNA Sequencing Analysis of YQHXJDD ELNVs. **(A)** Statistical analysis of miRNA length distribution; **(B)** Analysis of co-expressed miRNAs among samples; **(C)** Expression profiles of the top 20 most abundant miRNAs in YQHXJDD ELNVs.

The length distribution of small RNAs in three YQHXJDD ELNVs samples was analyzed (Figure 5A). The sequencing lengths of small RNAs in all three samples were predominantly concentrated within the range of 20–22 nt, which is consistent with the typical length characteristic of miRNAs. A Venn diagram (Figure 5B) was constructed to illustrate the co-expressed small RNAs among the three YQHXJDD ELNVs samples. A total of 514 small RNAs were co-expressed across all three samples, while each sample also exhibited unique small RNA expression patterns. The top 20 co-expressed small RNAs (miRNAs) in YQHXJDD ELNVs were listed in Figure 5C, providing detailed information including miRNA name, total counts, counts in each sample, length, and sequence. Notably, miRNAs such as miR8783 showed relatively high expression levels across the samples, with total counts reaching 14098.

### 4.6 Pharmacodynamics results

#### 4.6.1 *In Vivo* imaging

**FIGURE 6:**
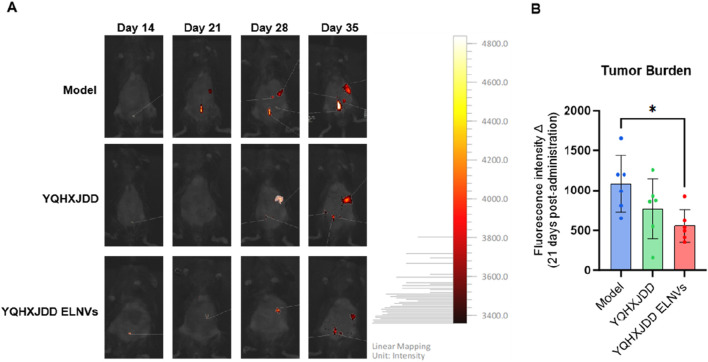
Changes in Tumor Burden-Related Fluorescence Intensity in Mice Following 3 Weeks of Treatment, as Determined by *In Vivo* Imaging. **(A)** *In vivo* imaging of mice; **(B)** Quantitative analysis of fluorescence intensity in murine tumor tissues; Compared with the model group: *P < 0.05.

Tumor burden was assessed by fluorescence intensity via *in vivo* imaging. Following 21 days of intervention, the YQHXJDD ELNVs group exhibited a significant inhibition of tumor growth compared with the model group (*P* < 0.05) (Figure 6).

#### 4.6.2 Omentum immunefluorescence intensity

**FIGURE 7:**
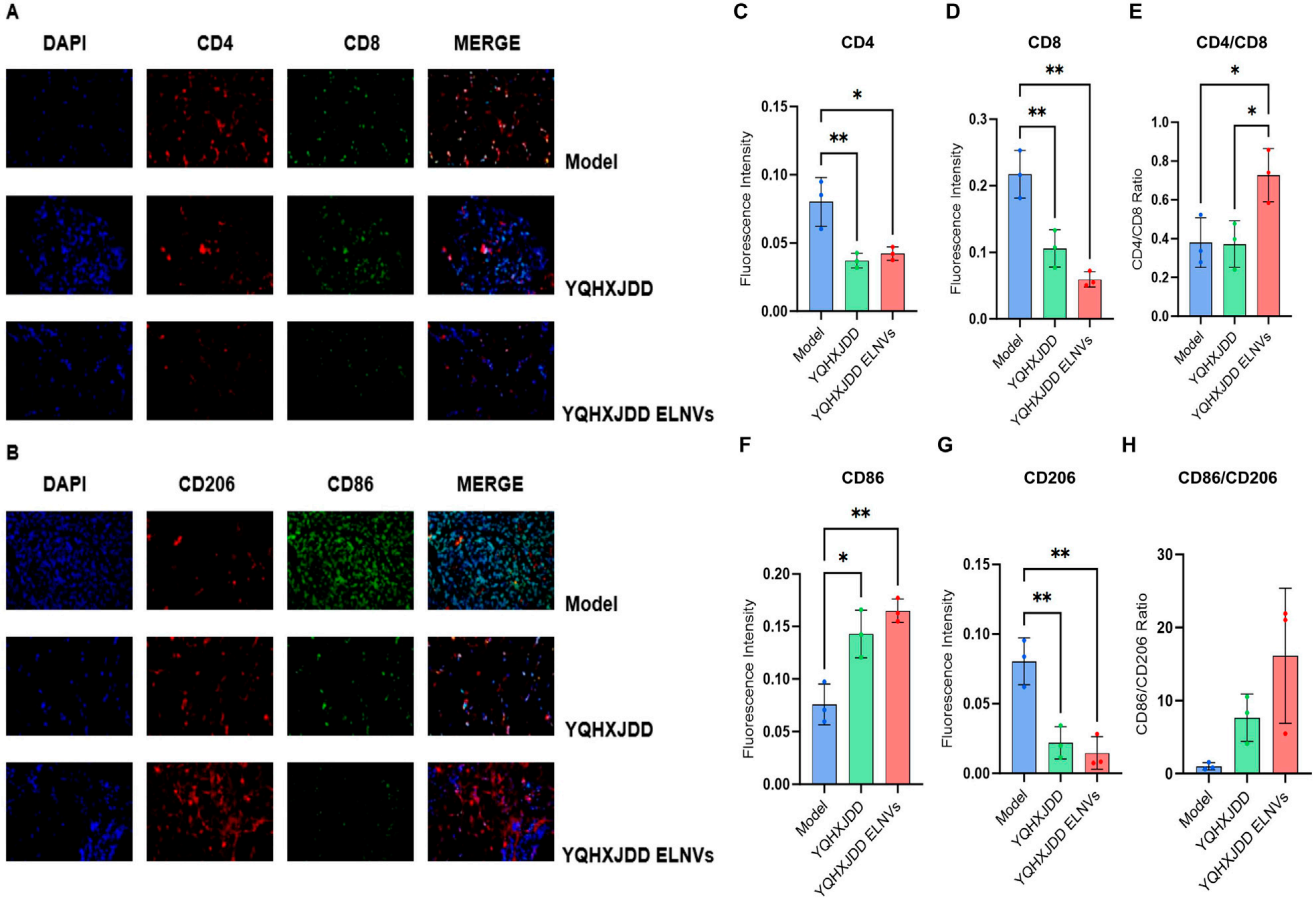
Immunofluorescence Analysis of Omental Tumor Tissues. **(A)** Immunofluorescence micrographs of CD4 and CD8 in murine omental tumor tissues (×400 magnification); **(B)** Immunofluorescence micrographs of CD206 and CD86 in murine omental tumor tissues (×400 magnification); **(C)** Quantitative analysis of CD4 fluorescence intensity in murine omental tumor tissues; **(D)** Quantitative analysis of CD8 fluorescence intensity in murine omental tumor tissues; **(E)** CD4/CD8 fluorescence intensity ratio; **(F)** Quantitative analysis of CD86 fluorescence intensity in murine omental tumor tissues; **(G)** Quantitative analysis of CD206 fluorescence intensity in murine omental tumor tissues; **(H)** CD86/CD206 fluorescence intensity ratio; Compared with the model group: *P < 0.05, **P < 0.01.

Omental tissues were sectioned, and the fluorescence intensities of CD4, CD8, CD206, and CD86 were evaluated (Figure 7). Compared with the model group, both the YQHXJDD ELNVs and YQHXJDD groups exhibited significantly higher CD86 expression (*P* < 0.05 and *P* < 0.01, respectively), markedly reduced CD206 fluorescence intensity (*P* < 0.01 for both groups). The YQHXJDD ELNVs group showed a significantly higher CD4/CD8 fluorescence intensity ratio compared with both the model group and the YQHXJDD group (*P* < 0.05).

#### 4.6.3 Cytokine and Chemokine analysis

**FIGURE 8:**
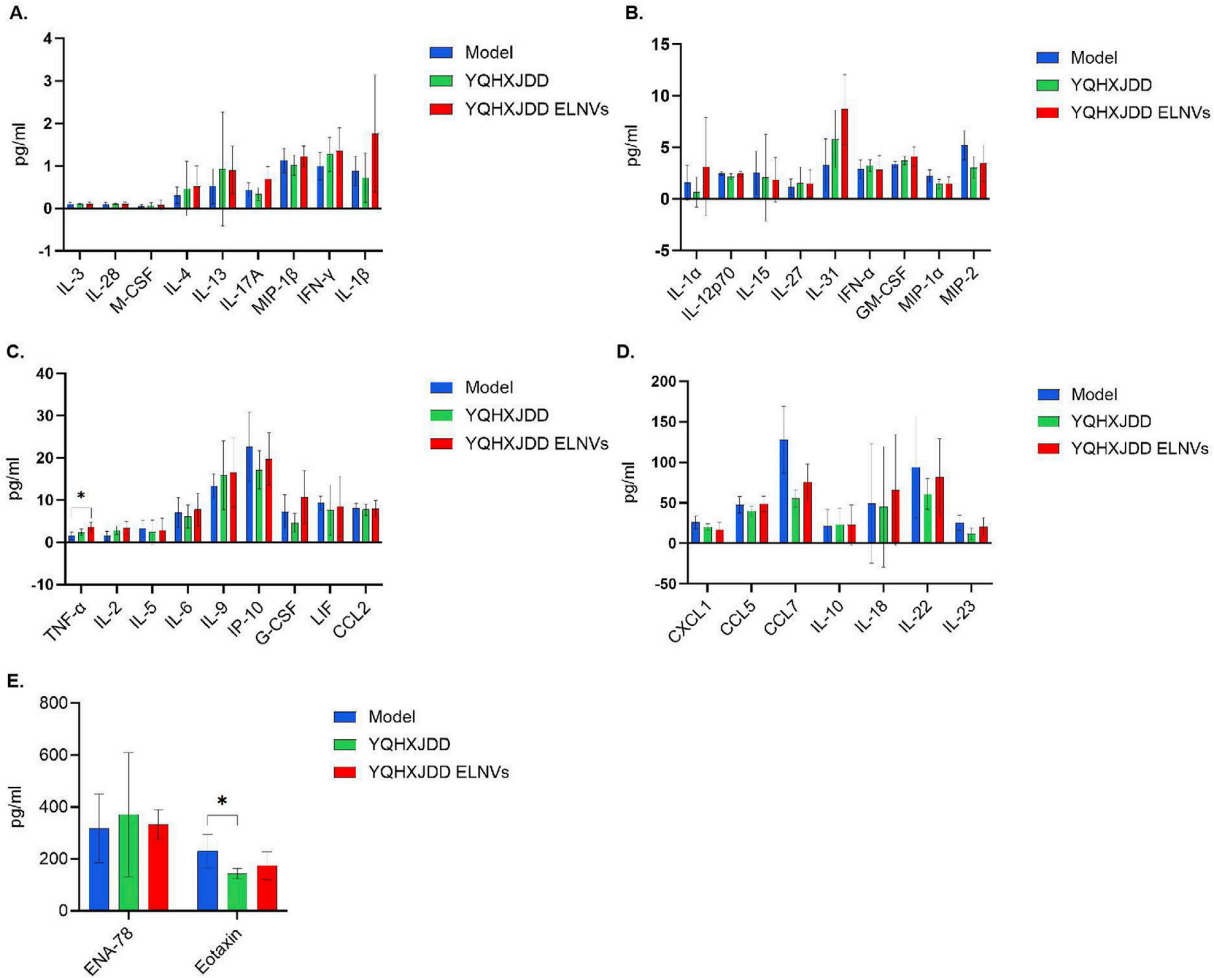
Group Differences in 36 Cytokines and Chemokines. **(A)** Differences in 8 cytokines and 1 chemokine (interleukin-13 [IL-13], interleukin-28 [IL-28], macrophage colony-stimulating factor [M-CSF], interleukin-4 [IL-4], interleukin-17A [IL-17A], macrophage inflammatory protein-1β [MIP-1β], interferon-γ [IFN-γ], interleukin-1β [IL-1β]) in murine peripheral blood; **(B)** Differences in 7 cytokines and 2 chemokines (interleukin-1α [IL-1α], interleukin-12p70 [IL-12p70], interleukin-15 [IL-15], interleukin-27 [IL-27], interleukin-31 [IL-31], interferon-α [IFN-α], granulocyte-macrophage colony-stimulating factor [GM-CSF], macrophage inflammatory protein-1α [MIP-1α], macrophage inflammatory protein-2 [MIP-2]) in murine peripheral blood; **(C)** Differences in 7 cytokines and 2 chemokines (tumor necrosis factor-α [TNF-α], interleukin-2 [IL-2], interleukin-5 [IL-5], interleukin-6 [IL-6], interleukin-9 [IL-9], interferon-γ-induced protein 10 [IP-10], granulocyte colony-stimulating factor [G-CSF], leukemia inhibitory factor [LIF], C-C motif chemokine ligand 2 [CCL2]) in murine peripheral blood; **(D)** Differences in 4 cytokines and 3 chemokines (C-X-C motif chemokine ligand 1 [CXCL1], C-C motif chemokine ligand 5 [CCL5], C-C motif chemokine ligand 7 [CCL7], interleukin-10 [IL-10], interleukin-18 [IL-18], interleukin-22 [IL-22], interleukin-23 [IL-23]) in murine peripheral blood; **(E)** Differences in 2 chemokines (epithelial neutrophil-activating peptide-78 [ENA-78], eotaxin) in murine peripheral blood; Compared with the model group: *P < 0.05.

Group differences in 36 cytokines and chemokines were presented in Figure 8. The level of tumor necrosis factor-α (TNF-α) in the YQHXJDD ELNVs group was significantly higher than that in the model group (*P* < 0.05). The level of Eotaxin in the YQHXJDD group was significantly lower than that in the model group (*P* < 0.05).

#### 4.6.4 Flow cytometric analysis of splenic immune cell subsets

**FIGURE 9:**
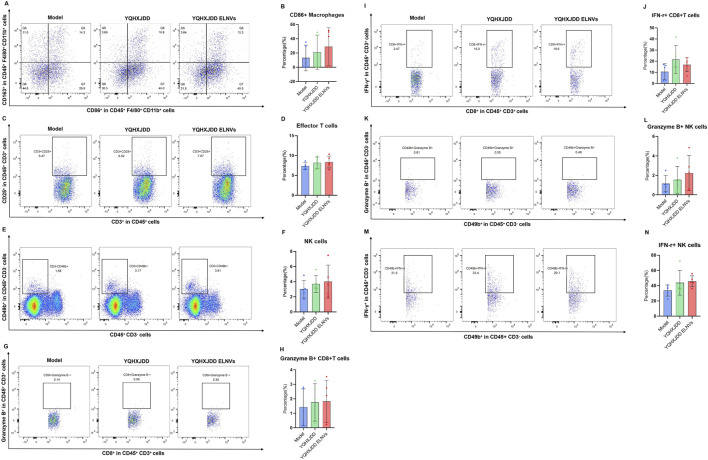
Splenic immune cell subsets. **(A)** Representative flow cytometry plots of M1-like and M2-like macrophages in different groups; **(B,D,F,H,J,L,N)**: Quantified percentages of CD86^+^ macrophages, effector T cells, NK cells, Granzyme B^+^CD8^+^ T cells, IFN-γ^+^CD8^+^ T cells, Granzyme B^+^ NK cells, and IFN-γ^+^ NK cells (n = 5); **(C,E,G,I,K,M)**: Representative flow cytometry plots of effector T cells, NK cells, Granzyme B^+^CD8^+^ T cells, IFN-γ^+^CD8^+^ T cells, Granzyme B^+^ NK cells, and IFN-γ^+^ NK cells in different groups.

The splenic immune cell subsets detected by flow cytometry were shown in Figure 9. Compared with both the YQHXJDD group and the model group, the YQHXJDD ELNVs group had no significant effect on the proportion of M1 macrophages (CD86^+^CD163^−^CD45^+^F4/80^+^) within the total macrophage population (CD45^+^F4/80^+^), effector T cells (CD25^+^CD3^+^CD45^+^) among total T cells (CD45^+^CD3^+^), and natural killer (NK) cells (CD49b^+^CD3^−^CD45^+^) within the CD3^−^CD45^+^ cell population. Additionally, relative to the model group, the YQHXJDD ELNVs-treated mice had no significant effect on the percentage of Granzyme B^+^CD8^+^ T cells among total CD8^+^ T cells, IFN-γ^+^CD8^+^ T cells among total CD8^+^ T cells, Granzyme B^+^ NK cells within the total NK cell population, and IFN-γ^+^ NK cells within the total NK cell population.

## 5 Discussion

Traditional Chinese medicines (TCMs) are diverse in types and medicinal parts, including dried aerial parts, roots, stems, leaves, seeds, barks, flowers, fruits, and beyond. In consideration of their physical properties, fresh plant-derived TCMs, characterized by high moisture content, allow for the extraction of bioactive constituents via techniques such as juicing and pulverization (Cao et al., 2019; Zhao et al., 2024). Conversely, dried medicinal materials necessitate processes like decoction to facilitate the dissolution and release of their active ingredients (Junyan et al., 2023; Du et al., 2019). Research on the extraction protocols for ELNVs has predominantly focused on single herbs, with relatively limited investigations into ELNV extraction from TCM compound formulations (Yu et al., 2020). The pharmaceutical ingredients of YQHXJDD are primarily dried medicinal materials, excluding fresh herbs and TCMs with high volatile oil content. Consequently, methods such as juicing, pulverization, or delayed addition (a technique where certain herbs are added later during decoction) are not utilized in the decoction process of YQHXJDD.

Currently, there is no unified standard for the decoction duration of dried Chinese herbal medicines, which require decoction to dissolve out their active substances, and the decoction time of most protocols is less than 1 h (Du et al., 2019). During the boiling step of TCM decoction, endogenous vesicles initially present in TCM raw materials first undergo disruption, followed by spontaneous reassembly to form novel vesicular structures (Yu et al., 2020). Notably, while a residual vesicle population persists post-boiling (a core procedural step in TCM decoction), these post-boiling vesicles are distinct from both the endogenous native vesicles inherently present in plant tissues and natural vesicles derived from other biological sources (Yu et al., 2020). Consequently, the boiling-induced transformation of herbal materials during decoction is indispensable for the biogenesis of these novel vesicles. Studies (Zhang et al., 2024; Li X. et al., 2019) have confirmed that ELNVs extracted from Chinese herbal decoctions after boiling exhibit good stability, and their bioactive components (e.g., lipids, small-molecule peptides) can still retain biological activity. Additionally, the newly formed bioactive substances generated during the decoction process are also crucial bioactive components of the TCM compound decoction and serve as the key to its therapeutic effects (Li T. et al., 2019). Our experimental findings also confirm that these boiling-derived vesicles exhibit favorable bioactive properties and exert potent therapeutic efficacy in relevant biological contexts. In optimizing the decoction parameters for extracting ELNVs from YQHXJDD, our approach was guided by two primary considerations: A) the risk of ELNV degradation induced by prolonged decoction; and B) the insufficiency of short-duration decoction to achieve complete extraction of bioactive components. To maximize alignment with clinical administration practices and thereby ensure therapeutic efficacy, we adjusted the decoction durations under high and low heat by both shortening and extending them, building upon commonly used clinical decoction methods. Ultracentrifugation subsequent to decoction represented the most widely applicable method for extracting ELNVs from Chinese herbal medicines, with the added advantage of yielding higher purity (Mu et al., 2023). Transmission electron microscopy (TEM) results demonstrated that ELNVs extracted via six different decoction protocols all maintained their typical “cup-shaped” morphology, with no obvious rupture or aggregation. And the NTA results showed that the particle size distribution remained stable, which is consistent with the structural characteristics of ELNVs. These observations demonstrated favorable morphological characteristics of ELNVs. Through particle size and concentration analysis, we calculated the maximum yield of ELNVs extractable from each dose of YQHXJDD. Protocol 3 yields significantly more ELNVs, indicating that the creation efficiency of ELNVs formed during decoction (using Protocol 3) is the highest. In contrast, ELNV yields from Protocols 4–6 decreased progressively. Besides, zeta potential analysis showed that YQHXJDD ELNVs isolated by Protocol 3 had an average zeta potential of −35.52 mV. With an absolute value exceeding 30 mV, this indicates strong inter-particle electrostatic repulsion in YQHXJDD ELNVs, which inhibits aggregation and ensures good colloidal stability. Therefore, by balancing extraction efficiency and structural stability, Decoction Protocol 3 achieved the highest ELNV yield (7.504 × 10^13^ particles per dose). Moreover, Decoction Protocol 3 aligned with conventional clinical decoction practices (Wang et al., 2025), endowing it with greater translational value. Therefore, the ELNVs isolated using Decoction Protocol 3 were employed for zeta potential measurement, untargeted metabolomics analysis, small RNA sequencing, and animal administration experiments.

In the untargeted metabolomics analysis of YQHXJDD ELNVs, metabolite classification results showed that within the SuperClass category, “Lipids and lipid-like molecules” represented the most abundant group, accounting for 34.98% of the total metabolites. Under the more detailed Class category, “Carboxylic acids and derivatives” was the dominant subgroup, with a proportion of 11.66%. Small RNAs in YQHXJDD ELNVs were predominantly 20–22 nt, consistent with the canonical features of miRNAs (a major class of small RNAs). This confirms they are functionally active, typical small RNAs, excluding non-specific nucleic acid fragment interference. Notably, miR8783 and other miRNAs showed distinct high expression with high conservation and abundance across samples, supporting their role as core regulators of YQHXJDD ELNVs’ biological functions and prioritizing them for subsequent functional validation.

Pharmacodynamic evaluations in animal models revealed that YQHXJDD ELNVs exerted a significant inhibitory effect on tumor growth in murine ovarian cancer models, while YQHXJDD did not. These results indicate that efficacy becomes more prominent after extraction into ELNVs, yet this finding warrants validation in larger sample sizes. The therapeutic efficacy of raw decoctions might be unsatisfactory due to their complex composition and low bioavailability (He and Pu, 2022). In contrast, ELNVs enhanced the therapeutic effect by enriching active ingredients (Zhao et al., 2024).

Immunofluorescence analysis of omental tissues demonstrated that the YQHXJDD ELNVs group exhibited higher CD86 fluorescence intensity and lower CD206 fluorescence intensity compared to the model group. These observations indicate that YQHXJDD ELNVs can increase the proportion of M1-polarized macrophages while reducing M2-polarized macrophages in omental tissues, thereby potentiating the antitumor activity of macrophages. It has been well-documented that the infiltration levels of CD4^+^ and CD8^+^ T cells are typically elevated in tumor tissues relative to normal tissues (Huang et al., 2015). However, in the present study, both the YQHXJDD ELNVs and YQHXJDD groups showed weaker fluorescence intensities for CD4 and CD8 compared to the model group, which might be attributed to the more extensive omental tumor infiltration observed in the model group. A reduced CD4/CD8 ratio within tumor tissues is closely associated with tumor progression and immune evasion, accompanied by a marked enhancement of the immunosuppressive phenotype of the tumor microenvironment. Such a reduction can result from functional exhaustion of CD8^+^ T cells and an expansion of regulatory T cells (Tregs) within the CD4^+^ T cell compartment (Huang et al., 2015; Facciabene et al., 2012; Riazi Rad et al., 2015). Notably, the YQHXJDD ELNVs group displayed the highest CD4/CD8 ratio, suggesting that YQHXJDD ELNVs can alleviate the immunosuppressive tumor microenvironment and enhance T cell-mediated immune activity.

Among the 36 cytokines and chemokines analyzed, YQHXJDD ELNVs significantly upregulated the peripheral blood level of TNF-α, thereby potentiating the cytotoxic activity against malignant tumors. However, the YQHXJDD group exhibited substantial reduction in Eotaxin. It is well-documented that Eotaxin contributes to tumor progression by recruiting eosinophils to remodel the tumor microenvironment, with its elevated expression closely associated with adverse clinical outcomes (Blank et al., 2017). These observations suggest that YQHXJDD may ameliorate the tumor microenvironment through the modulation of Eotaxin levels. Flow cytometric analysis revealed that YQHXJDD ELNVs had no significant effect on the proportions of splenic M1-polarized macrophages (with pro-inflammatory and anti-tumor properties), NK cells, and effector T cells, as well as the expression of functional markers (Granzyme B, IFN-γ) in effector T cells and NK cells (all *P* ≥ 0.05).

The decoction duration utilized in this study carries certain reference significance. Nonetheless, due to variations in types and physicochemical properties of traditional Chinese medicines (TCMs), the extraction protocol for dried material-derived YQHXJDD ELNVs may not be universally applicable. For volatile oil-rich TCMs (e.g., *Cinnamomi Cortex*, *Menthae Herba*), shorter decoction times are needed (Nie et al., 2018), while fresh materials (e.g., *Phragmitis Rhizoma*) are better processed via juicing. Compared to TCM decoctions, ELNVs have a relatively simplified composition, and their active ingredients are more amenable to identification. Consequently, ELNVs are more suitable for high-concentration and precisely targeted drug delivery.

Although YQHXJDD ELNVs and YQHXJDD both showed no statistical difference in therapeutic efficacy when compared to each other, YQHXJDD ELNVs exhibited statistically significant superior efficacy versus the model group (whereas YQHXJDD did not). The difference in therapeutic efficacy between YQHXJDD and YQHXJDD ELNVs requires a larger sample size to be confirmed, and this also constitutes a direction for our future research.

## 6 Conclusion

The decoction YQHXJDD ELNVs isolated from YQHXJDD constitute the key bioactive component underpinning the therapeutic efficacy of YQHXJDD, as they exert potent inhibitory activity against the growth and dissemination of ovarian cancer. Importantly, the miRNAs and lipid components encapsulated within YQHXJDD ELNVs are presumed to be the critical mediators responsible for its therapeutic effects.

## Data Availability

The raw data supporting the conclusions of this article will be made available by the authors, without undue reservation.

## Abbreviations

TNF-α, tumor necrosis factor-α; IL, interleukin; M-CSF, macrophage colony-stimulating factor; MIP, macrophage inflammatory protein; IFN-γ, interferon-γ; IFN-α, interferon-α; GM-CSF, granulocyte-macrophage colony-stimulating factor; IP-10, interferon-γ-induced protein 10; G-CSF, granulocyte colony-stimulating factor; LIF, leukemia inhibitory factor; CCL, C-C motif chemokine ligand; CXCL, C-X-C motif chemokine ligand; ENA-78, epithelial neutrophil-activating peptide-78.

## References

[B1] Blank S. Nienhüser H. Dreikhausen L. Sisic L. Heger U. Ott K. (2017). Inflammatory cytokines are associated with response and prognosis in patients with esophageal cancer. Oncotarget 8, 47518–47532. 10.18632/oncotarget.17671 28537901 PMC5564583

[B2] Bray F. Ferlay J. Soerjomataram I. Siegel R. L. Torre L. A. Jemal A. (2018). Global cancer statistics 2018: GLOBOCAN estimates of incidence and mortality worldwide for 36 cancers in 185 countries. CA Cancer J. Clin. 68, 394–424. 10.3322/caac.21492 30207593

[B3] Cao M. Yan H. Han X. Weng L. Wei Q. Sun X. (2019). Ginseng-derived nanoparticles alter macrophage polarization to inhibit melanoma growth. J. Immunother. Cancer 7, 326. 10.1186/s40425-019-0817-4 31775862 PMC6882204

[B4] Cao Y. Tan X. Shen J. Liu F. Xu Y. Chen Y. (2024). Morinda Officinalis-derived extracellular vesicle-like particles: anti-osteoporosis effect by regulating MAPK signaling pathway. Phytomedicine 129, 155628. 10.1016/j.phymed.2024.155628 38663117

[B5] Dad H. A. Gu T. W. Zhu A. Q. Huang L. Q. Peng L. H. (2021). Plant exosome-like nanovesicles: emerging therapeutics and drug delivery nanoplatforms. Mol. Ther. 29, 13–31. 10.1016/j.ymthe.2020.11.030 33278566 PMC7791080

[B6] Du J. Liang Z. Xu J. Zhao Y. Li X. Zhang Y. (2019). Plant-derived phosphocholine facilitates cellular uptake of anti-pulmonary fibrotic HJT-sRNA-m7. Sci. China Life Sci. 62, 309–320. 10.1007/s11427-017-9026-7 28378154

[B7] Facciabene A. Motz G. T. Coukos G. (2012). T-regulatory cells: key players in tumor immune escape and angiogenesis. Cancer Res. 72, 2162–2171. 10.1158/0008-5472.CAN-11-3687 22549946 PMC3342842

[B8] Feng Z. Huang J. Fu J. Li L. Yu R. Li L. (2025). Medicinal plant-derived exosome-like nanovesicles as regulatory mediators in microenvironment for disease treatment. Int. J. Nanomedicine 20, 8451–8479. 10.2147/IJN.S526287 40620682 PMC12227098

[B9] Han B. Zheng R. Zeng H. Wang S. Sun K. Chen R. (2024). Cancer incidence and mortality in China, 2022. J. Natl. Cancer Cent. 4, 47–53. 10.1016/j.jncc.2024.01.006 39036382 PMC11256708

[B10] He C. Pu Y. Q. (2022). Research progress on phase difference analysis of Chinese materia medica compound decoctions. Chin. J. Exp. Tradit. Med. Formul. 28, 259–266.

[B11] Huang Y. Ma C. Zhang Q. Ye J. Wang F. Zhang Y. (2015). CD4+ and CD8+ T cells have opposing roles in breast cancer progression and outcome. Oncotarget 6, 17462–17478. 10.18632/oncotarget.3958 25968569 PMC4627321

[B12] Hurwitz L. M. Pinsky P. F. Trabert B. (2021). General population screening for ovarian cancer. Lancet 397, 2128–2130. 10.1016/S0140-6736(21)01061-8 33991478

[B13] Junyan L. Wenping W. Yi Z. Zhizhong Y. (2023). Research progress on vesicles from Chinese medicinal herbs. Zhejiang da xue xue bao. Yi xue ban. 52, 349–360. 10.3724/zdxbyxb-2022-0715 37476946 PMC10409899

[B14] Li Y. F. Li J. Lu W. P. (2018). Effects of Yiqi huoxue jiedu formula on proliferation of ID-8 ovarian cancer cell line and angiogenesis process in mice. Chin. J. Exp. Tradit. Med. Formul. 24, 154–159.

[B15] Li X. Liang Z. Du J. Wang Z. Mei S. Li Z. (2019). Herbal decoctosome is a novel form of medicine. Sci. China Life Sci. 62, 333–348. 10.1007/s11427-018-9508-0 30900166

[B16] Li T. Wang P. Guo W. Huang X. Tian X. Wu G. (2019). Natural berberine-based Chinese herb medicine assembled nanostructures with modified antibacterial application. ACS Nano 13, 6770–6781. 10.1021/acsnano.9b01346 31135129

[B17] Li Y. Li J. Fan B. Wang Y. Jiang J. Zhang Z. (2020). Efficacy and safety of Yiqi Huoxue Jiedu decoction for the treatment of advanced epithelial ovarian cancer patients: a double-blind randomized controlled clinical trial. J. Tradit. Chin. Med. 40, 103–111. 32227771

[B18] Li J. Zhang Y. L. Jin T. Jin Z. Zhu M. Qing G. (2025). Advanced pharmaceutical nanotechnologies applied for Chinese herbal medicines. Adv. Sci. (Weinh) 12, e00167. 10.1002/advs.202500167 40538293 PMC12376714

[B19] Mu N. Li J. Zeng L. You J. Li R. Qin A. (2023). Plant-derived exosome-like nanovesicles: current progress and prospects. Int. J. Nanomedicine 18, 4987–5009. 10.2147/IJN.S420748 37693885 PMC10492547

[B20] Nie A. Z. Gao M. M. Zhu C. S. Zhang B. (2018). Discussion and reflection on special decoction methods of Chinese materia medica (II): Houxia (adding at a later stage). Chin. Tradit. Herb. Drugs 49, 3153–3161.

[B21] Peng Z. Gong Z. Wang Z. Deng B. Zhang X. Lin J. (2025). Salvia miltiorrhiza-derived exosome-like nanoparticles improve diabetic cardiomyopathy by inhibiting NLRP3 inflammasome-mediated macrophage pyroptosis via targeting the NEDD4/SGK1 axis. Nanomedicine (Lond) 20, 1417–1428. 10.1080/17435889.2025.2506351 40391625 PMC12143680

[B22] Riazi Rad F. Ajdary S. Omranipour R. Alimohammadian M. H. Hassan Z. M. (2015). Comparative analysis of CD4+ and CD8+ T cells in tumor tissues, lymph nodes and the peripheral blood from patients with breast cancer. Iran. Biomed. J. 19, 35–44. 10.6091/ibj.1289.2014 25605488 PMC4322231

[B23] Rutter B. D. Innes R. W. (2017). Extracellular vesicles isolated from the leaf apoplast carry stress-response proteins. Plant Physiol. 173, 728–741. 10.1104/pp.16.01253 27837092 PMC5210723

[B24] Wang P. Guo W. Huang G. Zhen J. Li Y. Li T. (2021). Berberine-based heterogeneous linear supramolecules neutralized the acute nephrotoxicity of aristolochic acid by the self-assembly strategy. ACS Appl. Mater Interfaces 13, 32729–32742. 10.1021/acsami.1c06968 34247476

[B25] Wang F. X. Chen F. W. Shen C. Y. Yue P. F. Shen B. D. (2025). Research progress on the scientific connotation of decoction methods for Chinese materia medica decoctions. Chin. J. Chin. Mater Med. 50, 994–999. 10.19540/j.cnki.cjcmm.20241031.301 40350817

[B26] Wei C. Chen Y. Chen J. Cao F. Cheng J. Pan C. (2025). miR166u -enriched Polygonatum sibiricum exosome-like nanoparticles alleviate colitis by improving intestinal barrier through the TLR4/AKT pathway. Int. J. Biol. Macromol. 318, 144802. 10.1016/j.ijbiomac.2025.144802 40451355

[B27] Weng J. Lu W. (2020). Exploration of Lu Wenping's thinking on treating ovarian cancer based on Piao Bingkui's academic thoughts. Int. J. Trad. Chin. Med. 42, 70–73.

[B28] WenJing R. Zx L. F Q. (2022). Research progress on traditional Chinese medicine affecting tumor therapy based on exosomes. Chin. Traditional Herb. Drugs 53, 7234–7241.

[B29] Wu X. Q. (2025). Clinical and mechanistic Study on the regulation of omental TAMs phenotype by Yiqi Huoxue Jiedu Fang to reverse platinum resistance in ovarian cancer. China Acad. Chin. Med. Sci.

[B30] Yi C. Lu L. Li Z. Guo Q. Ou L. Wang R. (2025). Plant-derived exosome-like nanoparticles for microRNA delivery in cancer treatment. Drug Deliv. Transl. Res. 15, 84–101. 10.1007/s13346-024-01621-x 38758499

[B31] Yu L. Deng Z. Liu L. Zhang W. Wang C. (2020). Plant-derived nanovesicles: a novel form of nanomedicine. Front. Bioeng. Biotechnol. 8, 584391. 10.3389/fbioe.2020.584391 33154966 PMC7591720

[B32] Zhang L. He F. Gao L. Cong M. Sun J. Xu J. (2021). Engineering exosome-like nanovesicles derived from Asparagus cochinchinensis can inhibit the proliferation of hepatocellular carcinoma cells with better safety profile. Int. J. Nanomedicine 16, 1575–1586. 10.2147/IJN.S293067 33664572 PMC7924256

[B33] Zhang Y. L. Wang Y. L. Yan K. Li H. Zhang X. Essola J. M. (2024). Traditional Chinese medicine formulae QY305 reducing cutaneous adverse reaction and diarrhea by its nanostructure. Adv. Sci. (Weinh) 11, e2306140. 10.1002/advs.202306140 38044276 PMC10837375

[B34] Zhao Q. Wang T. Wang H. Cao P. Jiang C. Qiao H. (2024). Consensus statement on research and application of Chinese herbal medicine derived extracellular vesicles-like particles (2023 edition). Chin. Herb. Med. 16, 3–12. 10.1016/j.chmed.2023.11.002 38375050 PMC10874762

